# Health plan adaptations to a mailed outreach program for colorectal cancer screening among Medicaid and Medicare enrollees: the BeneFIT study

**DOI:** 10.1186/s13012-020-01037-4

**Published:** 2020-09-15

**Authors:** Gloria D. Coronado, Jennifer L. Schneider, Beverly B. Green, Jennifer K. Coury, Malaika R. Schwartz, Yogini Kulkarni-Sharma, Laura Mae Baldwin

**Affiliations:** 1 Kaiser Permanente Center for Health Research, 3800 N. Interstate Ave., Portland, OR 97227 USA; 2 Kaiser Permanente Washington Health Research Institute, Seattle, WA USA; 3 Oregon Health & Science University, Portland, OR USA; 4 University of Washington Department of Family Medicine, Seattle, WA USA; 5 Molina Healthcare of Washington, Seattle, WA USA

**Keywords:** Medicaid, Colorectal cancer screening, Adaptations, Implementation, Direct-mail, Fecal immunochemical test (FIT)

## Abstract

**Background:**

Promoting uptake of evidence-based innovations in healthcare systems requires attention to how innovations are adapted to enhance their fit with a given setting. Little is known about real-world variation in how programs are delivered over time and across multiple populations and contexts, and what motivates adaptations.

**Methods:**

As part of the BeneFIT study of mailed fecal immunochemical tests (FIT) to increase colorectal cancer screening, we interviewed 9 leaders from two participating Medicaid/Medicare health insurance plans to examine adaptations to their health plan-initiated mailed FIT outreach programs in the second year of implementation. We applied an adaptation and modification model developed by Stirman and colleagues to document content and context modifications made to the two programs.

**Results:**

Both health plans made substantial changes to their programs in the second year; adaptations differed substantially across health plans. In Health Plan Oregon, adaptations generally targeted health centers and member populations, most content adaptations involved tailoring program components, and the program was expanded to four additional health centers. In contrast, Health Plan Washington’s second-year content adaptations were primarily at the level of members, and generally involved adding program components. Moreover, Health Plan Washington undertook large-scale context adaptations to the setting where the program was led (local vs. national), the personnel who administered the program (vendor and staffing), and the population selected for outreach (limiting outreach to dual-eligible members).

**Conclusions:**

Both programs implemented a variety of adaptations that reflected the values and incentives of the broader health plan contexts. Financial incentives for screening allowed Health Plan Oregon to expand but led Health Plan Washington to offer more targeted outreach to a subset of eligible enrollees. The breadth of changes made by each health system reflects the necessity of evaluating programs in context and adjusting to specific challenges as they are identified. Further research is needed to understand the effects of these types of adaptations on program efficiency and enrollee and health system outcomes.

Contributions to the literature

- Our data demonstrate the fluid nature of implementation of quality improvement programs within health plans: both health plans made numerous changes after the first year of the program in response to the realities of implementing the mailed fecal test program in their specific context.
- The different adaptations of this cancer screening program by the Oregon and Washington health plans were largely driven by differences in incentives: Medicaid incentives for colorectal cancer screening allowed Health Plan Oregon to expand its program after year 1, while the lack of such incentives led Health Plan Washington to restructure its program to focus exclusively on dual-eligible members.
- Our study is the first to apply the modified Stirman et al. framework to examine real-world adaptations to a cancer screening program initiated and undertaken by health plans; there are few reports of health plan-led adaptations to cancer screening programs.

## Background

Improving uptake of evidence-based innovations in healthcare systems requires understanding how innovations are modified to enhance their fit within a given setting. There is often a mismatch between the population or setting for which a given innovation was originally developed and validated and a particular population or setting in which it will be used [1–3]. As a result, program planners rarely know whether a program can produce similar outcomes when implemented in their population or setting. Adaptations made after introducing an innovation may be an important way to enhance the innovation’s success within new contexts. Previous research about how adaptations are implemented over time in real-world practice is limited. Such research could offer practical and timely guidance on how to select and implement adaptations to interventions in the context of real-world practice.

In our pragmatic study of a mailed fecal immunochemical test (FIT) program to increase colorectal cancer screening (BeneFIT), we partnered with two Medicaid/Medicare health plans in Oregon and Washington to evaluate the effectiveness of mailing fecal testing kits for colorectal cancer (CRC) screening to patients who meet screening criteria as a strategy to improve CRC screening rates. The BeneFIT program provides an ideal opportunity to study program adaptation, as abundant research has shown that mailed FIT outreach improves rates of CRC screening, but that the magnitude of the effect can vary depending on how the program is implemented [4–10], and little is known about how such programs are adapted.

We sought to document real-world adaptations undertaken by the two health plans participating in BeneFIT. We applied an adaptation and modification classification system developed by Stirman and colleagues [11, 12] to categorize the adaptations to the programs, distinguishing between *context* modifications (changes to format, location, or personnel delivering the intervention) and *content* modifications (changes to the intervention materials, procedures, or delivery) and categorizing the goals and reasons for each adaptation. By documenting the types and reasons for adaptations, our findings can guide health plans to select appropriate components and undertake adaptations suited to their contexts [13]. Our findings can also inform future studies that evaluate the effects of different types of adaptations on outcomes.

## Methods

### Study setting

BeneFIT is a study of two mailed FIT outreach programs, designed and implemented by two health plans providing Medicaid and Medicare insurance coverage for enrollees in Washington State (“Health Plan Washington”) and Oregon (“Health Plan Oregon”) [14]. Health Plan Washington provides insurance for approximately 650,000 Medicaid and dual Medicaid-Medicare enrollees in Washington State. Health Plan Oregon provides insurance for Medicaid and Medicare enrollees (with most Medicare enrollees dually eligible for Medicaid) for about 220,000 enrollees in Oregon. Both plans provide full coverage for CRC screening and follow-up testing with no out-of-pocket costs. Enrollees in these health plans receive their care at a variety of settings (e.g., health centers/ provider groups) in Washington and Oregon. For their initial designs, Health Plan Washington selected an opt-out approach for the health systems, and Health Plan Oregon recruited health centers for participation [14]. In Washington, all settings with Health Plan Washington enrollees were included (with the exception of 3 health systems that opted out) and individual health centers were not involved in implementation. In Oregon, implementation was shared between the health plan and health centers, and six health centers (that operated 26 clinics) were selected for participation in the evaluation (see below for more details on the implementation models in each state). The program was evaluated across 2 years. The health plans designed and executed both the initial mailed FIT outreach programs and any adaptations implemented in year 2.

Our processes for recruiting health plans and selecting eligible plan enrollees have been described elsewhere [14]. Our evaluation spanned 2 program years; the present analysis focuses on qualitative interviews following year 2 of the programs, which focused on assessing modifications to the program during the second year of implementation. In Health Plan Oregon the years were consecutive—2016 and 2017. Health Plan Washington paused the program in the second chronological year due to organizational restructuring; thus, its program years were 2016 and 2018. Both programs were offered on top of any existing CRC screening efforts at the health center- or provider-level.

### Health plan intervention models

Detailed descriptions of the first-year implementation models are described elsewhere [14]. The initial mailed FIT programs were based in part on our research team’s STOP CRC study [15] and the Washington plan’s previous experience offering mailed FITs to Medicare enrollees. Based on this initial experience, each health plan designed and implemented their own mailed FIT program for the study. The health plans adapted their programs with minimal guidance from the research team; the research team led each program’s evaluation.

#### Health Plan Oregon

Health Plan Oregon’s screening program used a “collaborative” model in which the health plan and participating health centers worked together to implement mailed FIT outreach. Health plan staff generated lists of eligible enrollees and distributed the lists to health centers, whose staff had the option to remove the names of anyone who was not a current patient or who was not a candidate for CRC screening (e.g., had recently been screened). Health plan staff provided an updated list and FIT kits selected by the health center (two-sample Insure® or one-sample OC-Auto® by Polymedco) to a mail vendor. The vendor then mailed an introductory letter, a FIT kit, and a postcard reminder to the enrollees on the list (*n* = 2650). Three of the six health centers also delivered phone call reminders, and two other centers offered incentives ($25 gift cards) for returning the FIT. The program used bilingual (English and Spanish) materials featuring the logos of both the health plan and the participating health center. Enrollees mailed or dropped off completed FITs to their assigned health center, where staff placed laboratory orders, processed kits, communicated test results, and assisted enrollees who screened positive in completing follow-up colonoscopies, following standard health center procedures.

#### Health Plan Washington

Health Plan Washington used a “centralized” program model, in which the health plan was the primary entity carrying out the program, with minimal collaboration with health centers/provider groups. The year 1 program was coordinated by health plan staff working in the health plan’s national office and was delivered to its 8,551 Medicaid and dual-eligible Medicaid-Medicare enrollees in Washington state. A centralized vendor mailed bilingual (English and Spanish) introductory letters and FIT kits (two-sample Insure® by Clinical Genomics) and delivered live reminder phone calls to enrollees using a list generated by the health plan. Incentives were offered to dual Medicaid-Medicare enrollees who returned a FIT kit ($15 gift cards). Completed kits were sent to a centralized laboratory for processing. Test results were sent to the health plan and to the enrollee’s provider, who followed their usual procedures to follow-up on positive FITs. Health plan care coordinators called enrollees with positive mailed FIT results to recommend contacting their primary care provider to discuss results.

### Data collection and analysis

#### Data collection

We conducted qualitative telephone interviews with the health plan leaders and staff who were involved in implementing the mailed FIT program after the first and second years of implementation. Analyses of year 1 interviews, exploring the successes and challenges of initial implementation, were published previously [16]. For the interviews following the second year, which focused on modifications to the program following initial implementation (the focus of the present analyses), we spoke with all staff at each health plan who were involved in second-year execution of the mailed FIT programs. For Health Plan Washington, this included the local vice president of quality, the local director of quality, and the local quality program manager. For Health Plan Oregon, this included the senior manager of delivery system improvement, the population health supervisor, a project manager who coordinated program activities with the health centers and vendor, and three panel managers (health plan staff who enhance service delivery in primary care patient panels).

We developed a semi-structured, open-ended interview guide based on findings from the first year of implementation and input from the study team. We explored factors motivating the health plans to continue the program for a second year; changes made to program components and reasons for these changes; second-year successes and challenges; and considerations for continuing to adapt, grow, and maintain the program into subsequent years. We conducted all interviews by telephone; each lasted approximately 45 min.

#### Adaptation measures and analysis process

We conducted qualitative coding based on the modification frameworks developed by Stirman and colleagues, as this represents the most comprehensive framework for studying program modifications currently available in the literature. Our first round of coding was based on their initial framework, which included four primary domains in their initial adaptation framework: (1) who made the modification, (2) what was modified, (3) at what level of delivery the modification was made (e.g., organization, unit, provider, population, network system/community), and (4) the type or nature of context or content-level modifications (e.g., adding elements, removing elements, tailoring/refining) [11]. During the coding process, an updated version of the framework was published: the Framework for Reporting Adaptations and Modifications-Expanded (FRAME) [12], which added the following components: (5) when and how the modification was made, (6) whether the modification was planned/proactive or unplanned/reactive, (7) who determined that the modification should be made, (8) the extent to which the modification was fidelity-consistent, and (9) the reasons for the modification, including (a) the intent or goal (e.g., reach, engagement; feasibility/implementation; fit with recipients; effectiveness, outcomes; cost reduction; satisfaction) and (b) contextual factors that influenced the decision.

For our specific process, all second-year interviews were conducted, audio-recorded, transcribed, and content-analyzed by a researcher (JLS) with expertise in qualitative methods and who had established rapport and engagement with health plan leaders and staff from prior baseline and year one interviews. As part of the first step in the coding and summarizing process, JLS created topical summaries of interview data for each health plan focusing on types of adaptations made, and implementation successes and challenges. The topical summaries were shared with the core research team (GDC, BBG, LMB, MRS) for review and refinement. Next, with guidance from the core research team, JLS then categorized content and context adaptations—with content modification defined as “changes made to the intervention procedures, materials or delivery”, and context modifications as “changes made to delivery of program setting or location, personnel delivering program or different population...” [11] In this process we identified the modification level (e.g., setting, personnel, population), and for content-based adaptations, we categorized the nature of the modification (e.g., tailoring/ tweaking/ refining, adding elements, removing elements). This application of the initial framework was shared with the core research team for discussion. Following consensus, we then further applied elements of the updated FRAME framework (5–9 above) to categorize the modifications by the goal (e.g., reach/ engagement, feasibility, fit with recipients), and reason (e.g., funding policies, funding or resource allocation, social context) [12]. In the categorization process, we added one additional goal not easily grouped into existing model categories: *implementation efficiency*. Again, application of the updated FRAME elements was reviewed by the core research team for discussion and refinement. Additionally, we shared findings as part of a “member check” process with interviewees for feedback and agreement of interpretation [17–19]. This iterative process resulted in the findings presented in manuscript (Tables 1 and 2).

**Table 1:** Context-based adaptations implemented in the second-year mailed FIT program

Program design*	O/W	Goal(s) for adaptation							Reason(s) for adaptation					Health plan-initiated adaptations	Context modification level		
		Reach, engagement	Feasibility	Fit with recipients	Effectiveness, outcomes	Implementation efficiency	Reduce costs	Satisfaction	Funding policies (incentives)	Funding or resource allocation	Social context	Service structure	Available resources		Setting	Personnel	Population
**Population receiving program**	**W**	●			●		●		●	●				- Refocused program on dual-eligible beneficiaries in WA state**	●		●
**Program alignment with priorities/incentives**	**W**			●	●				●	●				- Focus on supporting quality goals, such as Medicare 5-STAR - Focus on Medicare enrollees as they have more accurate address and prior screening information	●		●
	**O**			●	●			●	●	●				- Included FIT return in broader patient incentive program within health plan for meeting a range of preventive care needs ($25)			●
	**O**	●		●	●				●	●				- Began to identify need to prioritize future outreach to health centers serving Medicare enrollees	●		
**Program leadership**	**W**		●			●					●			- Transferred full program effort to state health plan Quality Improvement (QI) department involved in year one (previously led by national office) - Retained some local state QI staff involved in year one/hired new QI director with responsibility to lead program efforts	●	●	
**Vendor to deliver program (for phone calls, letters, FITs)**	**W**		●			●	●	●				●	●	- Contracted with new vendor that offered more services, e.g., delivered outreach calls and communicated FIT results to members and primary care providers	●	●	
**Program expansion**	**O**		●		●			●				●	●	- Health plan provided three additional staff to support some health centers with chart review, reminder calls, and processing returned kits		●	
	**O**	●			●				●	●	●		●	- Expanded program to 4 additional health centers	●		●

*Rows with “W” indicate Health Plan Washington; rows with “O” indicate Health Plan Oregon

**Year one included Medicaid and Medicare patients across multiple state

**Table 2:** Content-based adaptations implemented in the second-year mailed FIT program for Health Plan Oregon and Health Plan Washington

Program component*	O/W	Goal(s) for adaptation							Reason(s) for adaptation						Health plan-initiated adaptations	Content modification level*					Nature of content modifications*					
		Reach, engagement	Feasibility/implementation	Fit with recipients	Effectiveness, outcomes	Implementation efficiency	Reduce costs	Satisfaction	Funding policies (incentives)	Service structure	Available resources	Time constraints	Billing constraints	Provider perception of intervention		Organizational (health plan)	Unit (health centers)	Provider	Population (enrollees)	Network systems (e.g., vendor) Community	Tailoring/tweaking/refining**	Adding elements	Removing elements	Re-ordering elements	Condensing timeline	Substituting elements
**Identify eligible adults and obtain accurate contact information**	**W**	●								●					- Vendor obtained updated contact and enrollment information from member, if needed				●	●		●		●		
	**O**	●									●				- Increased patient address review in health record and mailing list - Improved capture of prior colorectal screening	●			●		●					
**Select FIT type**	**W**			●	●			●		●					- Selected vendor that offered a 1-sample FIT kit (previous vendor used 2-sample kit)	●			●	●	●					●
**Deliver program components (letters, phone calls, FIT kits)**	**W**	●		●	●	●	●			●	●	●			- Vendor mailed introductory letter and attempted up to 3 live calls to obtain patient permission to mail kit				●	●		●		●		
															- Vendor mailed FITs only to interested members, reached through live call				●	●			●			
															- Health plan mailed invitation letter to members having no phone number, asking them to contact the plan to participate	●			●				●			
	**O**	●			●	●					●			●	- Sent introductory letters (no FIT kit) to patients with no assigned provider or no clinic visits in last 12 months	●	●		●		●				●	●
	**O**	●			●				●		●	●			- Increased delivery of FIT reminders by health center staff (sometimes prioritized to Medicare enrollees)		●		●		●	●				
**Communicate test results**	**W**			●	●	●		●		●	●			●	- Vendor mailed result letter to member (normal and abnormal)				●	●		●			●	
															- Vendor concurrently informed PCP of abnormal result so PCP could order follow-up colonoscopy			●		●		●			●	
															- Vendor called enrollees with abnormal FIT results				●	●	●					●
**Streamline implementation**	**O**					●	●	●			●	●	●		- Directly obtained kits from lab on behalf of clinics - Improved vendor billing process	●	●			●	●				●	
	**O**	●			●	●					●	●			- Conducted a one-time (rather than split) mailing per health center	●	●		●		●				●	●

*Rows with “W” indicate Health Plan Washington; rows with “O” indicate Health Plan Oregon

#### Motivations for year-two adaptations

For each health plan, we present summaries and illustrative quotes from health plan staff about their motivations for continuing BeneFIT for a second year and a summary and perceived impact of second-year adaptations, and summarize the health plans’ ideas about further sustaining the programs.

#### Changes in screening rates

Our study was not designed to assess the merit of each plan’s adaptations. However, in the interest of completeness, we report changes in CRC screening rates following the second-year adaptations based on claims data from eligible enrollees.

## Results

All modifications were made after the year 1 implementation of the program and in the planning stage of year 2. Further, all modifications were planned and the need for modifications was determined by health plan staff. Because both health plans delivered the core intervention component, mailing of the FIT, over the 2 years, all adaptations were considered fidelity-consistent.

### Motivations for continuing BeneFIT for second year

Both health plans identified similar reasons for continuing the mailed FIT program for a second year. These included CRC screening continuing to be a high priority goal for the plans with the need to meet both state and national metrics (e.g., Oregon Medicaid incentive program and Medicare Five-Star Quality Rating System), strong support of the program from state-level quality improvement leaders within both plans, and a belief that the first-year results and successful implementation warranted its continuation for a second year. Additionally, both plans appreciated their partnership with the research team, which provided ongoing guidance and support to refine and implement the mailed FIT program.

*And the reason we were going to continue it was because we had partners in the research grant - and we love the partnerships…And it only makes sense when [the program] is set out to improve what we need to improve anyway…And I didn't see the [year one] data show that it wasn't successful enough to not want it again. But I also felt like over that first year I had made a lot of inroads with the provider groups…So, I think that's why we decided to continue.*—Health Plan Washington

*This is an area of clinical quality that we are held accountable for, so given that it is both a Coordinated Care Organization [state] incentive measure and a Medicare STARS measure, that plays a huge role in how we prioritize our efforts around those areas…in addition, colorectal cancer screening has been identified as an area of highest priority for our Medicare line of business in particular…Combined with the fact that we have seen a fairly good response from this program. So, it’s not just a strategy we put in place that kind of like crumbled and fell away and didn’t work. It appears to be working for our members in terms of the return rates that we’re seeing.*—Health Plan Oregon

### Second-year adaptations and perceived impacts—Health Plan Washington

Health Plan Washington leaders reported dramatic context adaptations in setting, personnel, and populations for the second-year (2018) implementation of the program (Table 1). At the national office, the health plan decided to discontinue its national outreach to Medicare enrollees across multiple states because CRC screening improvement was lower than anticipated, and the health plan experienced turnover in key national leadership roles. Given this turnover and the success of the research partnership developed in year 1, coordination of the program was shifted from the national office to regional health plan staff. The targets of the program also changed from all Medicaid enrollees to dual-eligible Medicaid-Medicare enrollees only (*n* = 1906), to focus on Medicare quality goals, and because these enrollees tended to have more accurate address and prior screening information. The program contracted with a new centralized outreach vendor that offered more services. These changes were motivated by funding policies (e.g., available incentives for Medicare enrollees), social context at the regional level (e.g., available staffing), and available resources.

With these context changes came several content adaptations as well (Table 2), including changes in how the program was delivered. The new process was to mail an introductory letter (in English) and deliver a live phone call to eligible enrollees on a list generated by the health plan, and only mail FIT kits to enrollees who agreed to receive the kit instead of mailing FIT kits to all eligible enrollees. The health plan also used a new FIT type (one-sample OC-Light®, Polymedco), and followed up with live telephone reminders after FITs were mailed (up to 3 attempts). Larger incentives ($40 gift cards rather than $15 gift cards) were offered to all enrollees who obtained CRC screening before the end of the year. Finally, test results were delivered to the health plan, the enrollee’s provider, and the enrollee at the same time. Providers followed their usual procedures to follow-up on positive FITs. Enrollees whose FIT results were positive were called by vendor staff, who provided results and recommended that they follow-up with their primary care provider. Content adaptations were driven by changes in service structure (e.g., services offered by the vendor), resource and time constraints, and provider perception of the program (e.g., desire for notification about abnormal test result). Most content adaptations in the Washington program were at the level of the health plan, enrollees and vendor and involved *adding* program components with the goal of improving the program’s effectiveness and enhancing the program’s fit with recipients.

Health Plan Washington perceived their second-year adaptations as helpful for program implementation. Moving implementation to the state-level allowed the health plan to be more nimble and strategic in addressing problems as they arose and enabled local staff to help track data. Partnering with a new vendor allowed the health plan to offer a one-sample FIT kit and more intensive outreach workflows (e.g., calls to obtain permission to send FIT kits first), which the health plan expected would improve member engagement with CRC screening. Finally, mailing FIT kits only to those members reached and interested was perceived as more efficient and aligned with member preferences:

*We put that [calls asking permission to mail FITs] in place simply because we want to make sure we’re reaching the member at the right time, and also updating our system - capturing the right contact information…And also, it’s easier in that I think members appreciate a heads up, rather than all of a sudden mailing it out with just a warning letter. It could get lost in the mail. And then it’s worked out well because at least they’re [members] expecting it… so they’re able to respond a little bit quicker.*—Health Plan Washington

### Second-year adaptations and perceived impacts—Health Plan Oregon

For the Health Plan Oregon program, leaders noted that the same general processes were followed in the second year as in the first year. The program was managed by the same staff and used the same mailed FIT vendor. Context changes (Table 1) in the Oregon program included expansion of the program to four new health centers, additional health plan staff to assist with delivering program elements and streamlining aspects of the implementation (processing FITs and vendor billing). The program also started to be integrated into a broader patient incentive program, offering $25 incentives for all enrollees, and focused efforts for program expansion to health centers with more Medicare enrollees. As in Health Plan Washington, these context changes reflected funding policies and resource allocation (e.g., incentives), social context (e.g., relationship with clinics), service structure (e.g., health plan staff to support clinics), and available resources.

Most content adaptations (Table 2) in the Oregon program were at the level of health centers and enrollees and involved *tailoring* program components with the goal of improving the program’s effectiveness and enhancing health center and enrollee engagement/reach. Adaptations included improving review of addresses and prior CRC screening. The health plan adaptations also *substituted elements*, such as sending invitational letters with no FIT kit to enrollees with no recent clinic visits (i.e. patients who had not established care at the health center) and *condensed the timeline* of FIT mailings to one mailing (rather than two) per health center. Reasons for these adaptations included funding policies (e.g., available health plan incentives), resource and time constraints, and provider perception of the program (e.g., that enrollees with no recent clinic visit were unlikely to return a mailed FIT). These improvements were likely facilitated by consistent staffing and vendor relationships. Individual health centers also made a variety of adaptations (often with staffing support from the health plan), such as scrubbing eligibility lists, delivering phone reminders, allowing FIT return by mail (versus dropping off at the health center), and providing patient incentives.

Interviewees from Health Plan Oregon expected that the second-year adaptations would both increase CRC screening rates and make the program more efficient. Additionally, supplying some health centers with health plan staff to support the program appeared to foster goodwill and ongoing collaborative relationships.

*I think the thing that really stuck out to us, was simply around list scrubbing and making sure that we were outreaching to the right members. And the fact that the clinics that went the extra step of then scrubbing those lists themselves and then sending out kits to a fewer number of people, we obviously saw better return rates.*—Health Plan Oregon

### Changes in screening rates following year 2 adaptations

For Health Plan Oregon, FIT completion rates rose 4.2% (from 17.4 to 21.6%) from year 1 to year 2 (data not shown) and any CRC screening rates increased 5.4% (from 19.6 to 25.0%; data not shown). For dual Medicaid-Medicare enrollees at Health Plan Washington, the two program implementation years yielded similar FIT completion rates (16.2% in 2016 vs 14.6% in 2018; data not shown) and any CRC screening rates (19.2% in 2016 vs 17.1% in 2018; data not shown).

### Sustainment of BeneFIT and future adaptations

At the time of the second-year interviews, each health plan was assessing whether their mailed FIT outreach program would be sustained in future years. For Health Plan Oregon, the success of the prior two years solidified the mailed FIT program as an ongoing service. With commitment from health plan leadership and dedicated staff funding for a project manager, they were planning a third year of the mailed FIT program. Staff were continuing to assess how to refine, integrate, and spread the program, with the following adaptations being considered for future implementation: offer the program to new health center partners; further refine the population targeted for the mail outreach, focusing on Medicare members; improve coordination with the broader health plan incentive program; improve member outreach/educational materials to include translation into additional languages; engage transitional or homeless members in partnership with community organizations; encourage health centers to routinely conduct practices to further improve return rates (e.g., scrubbing to improve accuracy of call list; phone reminders); and add more health plan staff to support more of health centers in scrubbing, delivering reminders, and processing returned kits. A leader from Health Plan Oregon summarized its experience with the program:

*If you look at the results that we’ve had … it’s getting better every year as we learn what works better in terms of micro-strategies that the clinics are using. And so I think that it’s something that I see us having the ability to fine-tune to allow for us to even increase our rates [in the future]…There were new clinics that came on - again, it seemed like something that we were seeing some success with and wanted to spread that across the system more…So for example, we hadn’t done a breakdown by line of business previously and we know that there are some systems that have higher percentages of our Medicare line of business. And so we’re… reaching out more and more to those clinics and those partners…and we’re being a little bit more deliberate about sharing the best practices and suggestions for how an individual clinic actually rolls out the program, the scrubbing, the follow up calls, things like that… We had information go out to members about different incentives that we were offering for different preventive services. And colorectal cancer screening was included.*—Health Plan Oregon

For Health Plan Washington, the future of the mailed FIT outreach program was less certain at the time of the interviews. The implementation staff continued to assess year 2 return rates against the cost of implementing the program. Even with this uncertainty, future adaptations were being considered, including refining workflows with the mailed FIT vendor to improve efficiency and accuracy in communication and tracking; establishing partnerships with provider groups to improve patient receptivity (such as a co-branded outreach letter from plan and provider); increasing and improving direct-to-member education on the importance of CRC screening and ease of mailed FIT options (e.g., improve outreach letter with more educational and motivational content); and tracking outcomes of positive FIT results and colonoscopy completion as part of the overall evaluation of the program. A Health Plan Washington leader commented on the program’s future:

*I think it’s sustainable because we track our members who need these tests on a monthly basis - really, it’s more about getting them to respond and helping them understand why it’s important… we need to do more prepping by giving the members a heads up, trying to coordinate it more with the providers if possible…helping with that piece of operations would be following up on any of the positive results with the providers. And that was sent out to them, but what came out of those positive results? So looking at more outcome based stuff – that is true value in terms of what we could actually do to the program. So I think tweaking a few of these things, and really knowing what’s happening on a weekly basis from the vendor on a more regular basis, would be immensely helpful.*—Health Plan Washington

## Discussion

Through qualitative interviews and application of the FRAME framework, we examined the adaptations made by two health plan-initiated mailed FIT outreach programs in the second year of operation. Both plans made extensive adaptations, ranging from wholesale changes in program implementation to fine-tuning of mailing and reminder procedures. The number and breadth of these adaptations illustrates the amount of modification needed even when beginning with an initiative designed by the health plan itself. The specific changes made reflect the unique context each health plan faced when planning a sustainable CRC screening program.

In Health Plan Oregon, adaptations were primarily changes in content, most targeting health centers and member populations and involving tailoring program components. These adaptations included changes to improve identification of eligible enrollees, changes to mailing procedures and instructions intended to improve engagement, and shifting of administrative burden from health centers to the health plan. These adaptations were intended primarily to improve processes for selecting eligible members. Context changes in the Health Plan Oregon program included expansion to four new health centers, which was possible largely because state-level Medicaid incentives for CRC screening allowed the health plan to dedicate resources to the program. Other adaptations worked to streamline operations so that the program could be as cost-effective as possible. Increases in screening rates in year 2 of the program suggest that the adaptations may have improved the program’s effectiveness, although more research is needed to rigorously assess the effects of these changes.

Content adaptations at Health Plan Washington were primarily at the level of members, and generally involved adding program components, with the goal of supporting member engagement and improving follow-up care. Health Plan Washington also undertook several large-scale context adaptations: they made adaptations to the setting where the program was led (state vs. national), the personnel who administered the program (vendor and staffing), and the population selected for outreach (dual-eligible members). The overarching goal of these context changes was to create a more nimble, flexible program that was locally managed and aligned with the incentives in place in the Washington context: specifically, health plans in Washington were only incentivized for screening Medicare enrollees (Medicare 5-Star Program), not Medicaid enrollees (health plans in Oregon were incentivized for both). Health Plan Washington was also motivated to improve the program’s return on investment and enhance its fit with recipients by implementing an opt-in approach, mailing a one-sample versus two-sample FIT kit, and sending a letter to enrollees with no phone number on file. While these changes did not result in higher screening rates in year 2 among Medicaid/Medicare enrollees, rates were relatively similar across years, and costs were reduced due to the opt-in format.

Our findings are consistent with Escoffrey and colleagues’ recent review of adaptations to 42 evidence-based interventions, which found that the most common types of content adaptations were tailoring (93% of studies reviewed) and adding elements (71% of studies reviewed) [20]. In that study, common reasons for adaptations were cultural appropriateness (64%), focusing on a new target population (60%), and implementing in a new setting (57%). In contrast, our adaptions were primary motivated by funding and resource policies and constraints, social context, and service structure, likely because our program was implemented over 2 years by the same health plans.

Both health plans also made adaptations to improve the identification of enrollees. While past research has shown that claims data can accurately identify prior CRC screening [21], our study identified additional aspects of patient identification that are necessary for a successful mailed FIT intervention: accurate contact information, and information about whether enrollees had established care at a given health center.

Both mailed FIT programs were designed to be implemented primarily at the health plan-level. While Health Plan Washington used a centralized model with minimal health center involvement beginning in year 1, Health Plan Oregon initially used a more distributed model, but centralized more program components in year 2. This is consistent with larger trends of Medicaid Managed Care plans and Medicare Advantage plans becoming increasingly involved in conducting direct-to-member outreach to promote uptake of CRC screening and other preventive health screenings [8]. This direct-to-member outreach can leverage some advantages over clinic-level efforts: it minimizes burden on clinics, facilitates cost-sharing between health plans and clinics, and creates efficiencies by standardizing processes across health centers and creating economies of scale when working with vendors. Health plan staff are also uniquely qualified to reassure patients about coverage for FIT and follow-up colonoscopies, as needed.

Our study shows that there are several ways in which health plans can deliver mailed FIT outreach to eligible enrollees. Our health plan initial implementation models were distinct, and each health plan made numerous adaptations in their second implementation year. Future research might compare the effectiveness, incremental effectiveness, and cost-effectiveness of selected and combined approaches, across a variety of settings.

### Strengths and limitations

This study had several strengths. There was little researcher involvement in adapting the programs for year 2, allowing us to naturalistically assess how health plans made changes. Interviews were conducted with all key health plan staff involved in the second-year implementation of the program, using established qualitative interviewing and analysis techniques. Our use of the standardized FRAME framework will enable comparisons with future studies and inform our broad understanding of program adaptations and facilitate implementation, scale-up, spread, and sustainment of evidence-based innovations.

This study also had several limitations. The scope of the analysis was limited to health plan-level adaptations—we interviewed health plan leaders and staff and did not consistently capture data from health centers and provider groups. We plan to explore health center adaptations in a future paper. It is possible that there were undocumented adaptations at these levels. Moreover, because the Washington health plan paused its program in 2017, the time frames for our second-year evaluations differed.

## Conclusion

Both health plans implemented a wide range of adaptations in the second year of their mailed FIT programs. Adaptations reflected the specific norms and priorities of each health plan’s broader context. Specifically, state Medicaid incentives for CRC screening allowed Health Plan Oregon to expand its program after year 1, while the lack of such incentives led Health Plan Washington to restructure its program to focus exclusively on dual-eligible members.

## Data Availability

The datasets used and/or analyzed during the current study are available from the corresponding author on reasonable request.

## Abbreviations

CRC: Colorectal cancer
FIT: Fecal immunochemical test
FRAME: Framework for Reporting Adaptations and Modifications-Expanded
